# Evaluation of inherited germline mutations in cancer susceptibility genes among pancreatic cancer patients: a single-center study

**DOI:** 10.1186/s10020-023-00600-1

**Published:** 2023-01-30

**Authors:** Francesca Tavano, Domenica Gioffreda, Andrea Fontana, Orazio Palmieri, Annamaria Gentile, Tiziana Latiano, Anna Latiano, Tiziana Pia Latiano, Matteo Scaramuzzi, Evaristo Maiello, Francesca Bazzocchi, Francesco Perri

**Affiliations:** 1 Division of Gastroenterology, Fondazione “Casa Sollievo della Sofferenza” IRCCS Hospital, Viale Cappuccini 1, FG 71013 San Giovanni Rotondo, Italy; 2 Unit of Biostatistics, Fondazione “Casa Sollievo della Sofferenza” IRCCS Hospital, Viale Cappuccini 1, FG 71013 San Giovanni Rotondo, Italy; 3 Department of Oncology, Fondazione “Casa Sollievo della Sofferenza” IRCCS Hospital, Viale Cappuccini 1, FG 71013 San Giovanni Rotondo, Italy; 4 Department of Surgery, Fondazione “Casa Sollievo della Sofferenza” IRCCS Hospital, Viale Cappuccini 1, FG 71013 San Giovanni Rotondo, Italy

**Keywords:** Pancreatic cancer, Genetic testing, Next Generation Sequencing, Germline variants, Prevalence, Cancer family history

## Abstract

**Background:**

Germline mutations in cancer susceptibility genes were identified in pancreatic cancer (PanC) patients with a sporadic disease and in those unselected for family cancer history.

**Methods:**

With the aim to determine the prevalence of germline predisposition genes mutations in PanC, and to evaluate whether they were associated with the presence of PanC, we profiled a custom AmpliSeq panel of 27 cancer susceptibility genes in 47 PanC patients and 51 control subjects by using the Ion Torrent PGM system.

**Results:**

Multigene panel testing identified a total of 31 variants in 27 PanC (57.4%), including variants with pathogenic/likely pathogenic effect, those of uncertain significance, and variants whose clinical significance remains currently undefined. Five patients carried more than one variant in the same gene or in different genes. Eight patients (17.0%) had at least one pathogenic/likely pathogenic variant in four main genes: CFTR (10.6%), BRCA2 (8.5%), ATM and CHEK2 (2.1%). Pathogenic/likely pathogenic mutation were identified in patients with positive PanC family history (20%) or in patients without first-degree relatives affected by PanC (13.6%). All the BRCA2 mutation carriers were unselected PanC patients. The presence of mutations in BRCA2 was significantly associated with an increased occurrence of PanC and with positive family history for endometrial cancer (p = 0.018).

**Conclusions:**

This study confirmed the potential remarkable contribution of BRCA2 in assessing the presence of PanC. Overall our findings supported the recommendation of offering the germline testing to all the PanC patients with the intent to reduce the number of underdiagnosed carriers of mutations in predisposition genes, and not to preclude their relatives from the opportunity to benefit from surveillance programs.

**Supplementary Information:**

The online version contains supplementary material available at 10.1186/s10020-023-00600-1.

## Introduction

Pancreatic cancer (PanC) is expected to become the second leading cause of cancer mortality by the year 2030 (American Cancer Society 2018). New strategies for risk assessment, screening, and treatment are urgently needed to render more patients eligible for early detection and curative resection, and to prolong the disease survival rate.

PanC is well characterized at molecular level. Somatic mutations in PanC occur in the oncogene KRAS, and in the tumor suppressor genes CDKN2A, TP53 and SMAD4 (Jones et al. 2008). Oncogenic alterations in ERBB2 cooperating with KRAS mutants to promote PanC tumorigenesis have been also identified (Li et al. 2020). Moreover, accumulation of mutations in GNAS and RNF43 genes has been reported to affect the normal functioning of Wnt pathway during the pancreatic carcinogenesis (Lee et al. 2016).

Both modifiable and genetic risk factors contribute to the development of PanC: typically associated environmental and lifestyle factors include smoking, obesity, alcohol abuse and diabetes; a hereditary component has been identified in approximately 10% of cases, with a specific germline mutation being implicated in 20% of those cases (Lowenfels and Maisonneuve 2006; Tsai and Chang 2019; Klein et al. 2001). Germline mutations in a growing number of genes have been associated with increased lifetime risks of PanC, including several cancer susceptibility genes that predispose to well-characterized cancer syndromes (Solomon et al. 2012; Klein 2012; Weiss 2014; Couch et al. 2005), (Table 1). In addition, germline mutations in CHEK2 have been found with a prevalence of 4% in patients with PanC (Mandelker et al. 2017), and a number of genetic alterations of the proofreading domain of DNA polymerases, such as POLE or POLD1, have been associated with the risk for human cancers (Barbari and Shcherbakova 2017).

**Table 1:** Syndromes and respective gene(s) associated with pancreatic cancer

Syndrome	Gene(s)
Hereditary breast and ovarian cancer	BRCA1, BRCA2, PALB2
Familial atypical multiple mole melanoma	CDKN2A
Lynch	MLH1,MSH2, MSH6, PMS2, EPCAM
Peutz-Jeghers	STK11
Hereditary pancreatitis	PRSS1, SPINK1, CFTR
Familial adenomatous polyposis	APC
Li-Fraumeni	TP53
Ataxia-telangiesctasia	ATM
Fanconi anemia	FANCC, FANCG

Recent studies have found that 4% to 25% of patients with apparent sporadic PanC (i.e., patients who do not have a family history of PanC or a family history suggestive of an inherited cancer syndrome) have germline mutations in a PanC susceptibility genes (Grant et al. 2015; Hu et al. 2018; Shindo et al. 2017). Germline mutations have also been observed in 7% of unselected PanC (i.e. patients who have a family history of PanC or other cancers but did not meet criteria for familial PanC or any cancer predisposition syndromes), (Hu et al. 2016). These findings underline the limitations of current guidelines for genetic testing in patients with PanC, according to which surveillance is reserved for members of a family with at least 2 first-degree relatives with PanC or for subjects at highest risk because of a known hereditary cancer syndrome associated with PanC. Thus, routine gene testing for inherited susceptibility is currently being suggested for all patients diagnosed with PanC (Shindo et al. 2017).

Herein, we used a custom gene panel to assess the prevalence of germline mutations in 27 susceptibility genes in a single center population including PanC patients and control subjects. Case–control analysis was performed to evaluate the association between inherited germline mutations and the presence of PanC. A particular attention was paid to evaluate how cancer family history and other risk factors can contribute to the PanC assessment. The aim was to establish a defined subset of genes that confer susceptibility to PanC, and will likely have the potential to assist clinicians in developing approaches for screening and detection of early asymptomatic patients and in managing PanC.

## Methods

### Study population

The study population was recruited at the Divisions of Gastroenterology, Oncology, and Surgery of Fondazione “Casa Sollievo della Sofferenza” IRCCS Hospital, San Giovanni Rotondo (Italy) from 2011 to 2018. The study was approved by the local Ethics Committee (Prot. No. 96/CE/2011). Participation consisted of completing a consent form and providing a venous blood sample. A total of 98 participants were enrolled into the study, including patients who received the diagnosis of PanC (No. = 47) and control subjects (CS, No. = 51). The control cohort included subjects with functional gastrointestinal disease (e.g., gastritis, gastro-esophageal reflux disease, dyspepsia, irritable bowel syndrome and somatic stress) attending the outpatient clinic of the Division of Gastroenterology. All the CS were retained into the study after being interviewed to unravel the presence of one or more clinical risk factors associated with the development of PanC, and after a clinical evaluation and ultrasound imaging disclosed a normal pancreas.

The risk factor survey in both PanC patients and controls collected information on age at disease diagnosis/sample collection (where patients diagnosed with the malignancy at less than 55 years old were considered early onset PanC), smoking habit, alcohol intake, body mass index (where subjects with an index greater than 30 kg/m^2^ were classified as obese), and diabetes (where if diabetes was ascertained within 2 years prior to enrollment into the study or to the PanC diagnosis, for controls and cancers cohorts, respectively, subjects were classified as having early-onset diabetes). Information about occurrence of either PanC and other cancers among first-degree relatives was also collected from both PanC patients and controls: family history for PanC, for cancers associated to the hereditary cancer syndromes (i.e. breast, ovarian, endometrial, colon cancer and melanoma), and for other cancers (i.e. cancers different from those associated to the hereditary cancer syndromes) including lung, gastric, prostate, head and neck, liver, bladder, adrenal cancer and leukemia were recorded; this information was used to classify patients into familial PanC (i.e. kindred with a pair of first-degree relatives affected by PanC), unselected PanC (i.e. patients with family history of PanC or other tumors suggestive of an inherited cancer syndrome, such as breast/ovary/colorectal/melanoma, but did not meet criteria for familial PanC or any cancer predisposition syndromes) or sporadic PanC (i.e. patients who do not have a family history of PanC or a family cancer history suggestive of an inherited cancer syndrome). Patients were also asked to authorize study team access to medical record in order to collect clinic-pathologic data including tumor location, tumor staging, type of chemotherapy, duration of therapy and response to treatment. Patients were prospectively followed until death or censoring (whichever occurred first) and the last information on vital status was obtained on 31 January 2021.

### Sample preparation

Blood was withdrawn from the peripheral veins into sodium citrate vacutainer tubes (BD Vacutainer®, BD, USA) and stored at -30 °C. Genomic DNA was extracted from peripheral venous blood by using the QIAamp DNA Blood Maxi Kit (Qiagen, Hilden, Germany) following the manufacturers’ recommendations.

### Next Generation Sequencing based on AmpliSeq panel and Ion Torrent PGM system

Genomic DNAs were tested by Next Generation Sequencing (NGS) using an Ion AmpliSeq Custom Panel (Cat. No. A35121, Thermo Fisher Scientific, Foster City, CA), designed with the Ion AmpliSeq Designer (https://www.ampliseq.com), to identify disease causing/associated mutations in 27 susceptibility genes. This design allowed analysis of 487 exons (padding: +/−25 bp) by the targeted resequencing of 775 amplicons (global size: 144,188 kb/patient). An amplicon library of the target exons was prepared with custom panel of target regions covering all coding regions and consensus splice sites from TP53 (NM_000546), STK11 (NM_000455), SMAD4 (NM_005359), PMS2 (NM_000535), MUTYH (NM_012222), MSH6 (NM_000179), MSH2 (NM_000251), MLH1 (NM_000249), CFTR (NM_000492), BRCA2 (NM_000059), BRCA1 (NM_007294), APC (NM_000038), CDKN2A (NM_000077), KRAS (NM_033360), GNAS (NM_000516), ATM (NM_000051), ERBB2 (NM_004448), CHEK2 (NM_001005735), EPCAM (NM_002354), SPINK1 (NM_003122), PRSS1 (NM_002769), FANCC (NM_000136), FANCG (NM_004629), MSH3 (NM_002439), RNF43 (NM_017763), POLD1 (NM_001256849), POLE (NM_006231).

#### Amplicon library preparation

Amplicon libraries were constructed using the Ion AmpliSeq™ Library Kit Plus (Cat. No. A35907, Thermo Fisher Scientific), and the Ion Xpress™ Barcode Adapter 1–96 Kit (Cat. No. 4471250, Thermo Fisher Scientific) according to the manufacturer's instructions. Briefly, 10 ng of DNA were used to generate the sequencing libraries with two pools of 385 and 390 primers, respectively: after target amplification in 10 µl reactions, pool 1 and pool 2 amplification reactions were combined. Library preparation resulted in a single sample library, and each sample library was assigned a barcode adapter to differentiate between samples. Barcoded libraries were ran on high sensitivity D1000 Screen Tapes (Cat. No. 5067-5584, Agilent Technologies, Santa Clara, CA) using the Tapestation 2200 (Agilent Technologies) to assess both the quality and the band size, and calculate the final library pool molarity. Eight sample libraries were then normalized to a concentration of 100 pM before they were combined for subsequent template preparation.

#### Emulsion PCR

Clonal amplification of the libraries was carried out by emulsion PCR performed by using the Ion OneTouch 200 Template Kit v2 (Cat. No. A29900, Thermo Fisher Scientific) on the Ion OneTouch™ System (Thermo Fisher Scientific) according to the manufacturer's instructions. Subsequently, enrichment for template-positive Ion Sphere™ Particles was achieved using the Ion PGM™ Enrichment Beads (Cat no. 4478525, Thermo Fisher Scientific) on the Ion OneTouch Enrichment System (Thermo Fisher Scientific), following the protocol recommended by manufacturers.

#### Sequencing

Enriched, templated beads were loaded onto an Ion Torrent 318 V2 chip (Cat. No. 4488146, Thermo Fisher Scientific) and sequenced using the Ion PGM Hi-Q View Sequencing kit (Cat. No. A30044, Thermo Fisher Scientific) on the Ion Torrent Personal Genome Machine (PGM) instrument (Thermo Fisher Scientific) according to the manufacturer's instructions. The Ion PGM performed 500 flows for each run.

#### Bioinformatics analysis

The Torrent Suite Software version 5.0.5 (Thermo Fisher Scientific) was used to process raw data acquired by Ion Torrent PGM instrument, and the Torrent Server was used to successively map the human genome sequence (build GRCh37/hg19) with a Torrent Mapping Alignment Program optimized to Ion Torrent™ data. After the sequence mapping, the DNA variant regions were piled up with Torrent Variant Caller plug-in software set to run at high stringency. Following the analysis, the annotation of single nucleotide variants and indels was performed using the Ion Reporter Server System version 5.6 (Thermo Fisher Scientific).

Data from Ion Reporter were filtered to exclude low frequency germline variations (allelic frequencies too far from 100% or 50%), variants located more than 10 bp upstream or downstream of the coding region, synonymous variants, all those with evidences of benign/likely benign effect or to a weak pathogenicity in ClinVar (http://www.ncbi.nlm.nih.gov/clinvar/) and Leiden Open Variation Database v.3.0 (LOVD; https://databases.lovd.nl), and those indicated as a risk factor by the literature.

Variants occurring in 100% of reads, or allele frequencies over 35% and up to 65% were considered. We further only paid attention to variants identified in less than 3 subjects. Germline variants (nonsense, missense, frameshift, canonical +/−1 or 2 splice sites, and insertion/deletion) with supporting evidence for variant classification and clinical impact were considered for the analysis, including variants with pathogenic or likely pathogenic effect, variant of uncertain significance (VUS), and those categorized as either pathogenic/likely pathogenic and VUS (Richards et al. 2015; Plon et al. 2008; Moghadasi et al. 2016). In addition, variants whose clinical significance currently remains undefined in curated databases for the interpretation of sequence variants (UVs: uncategorized variants) were also taken into account when occurring in subjects without other variations supported by a pathogenicity evidence.

### Validation by Sanger sequencing

NGS variant calls were validated by targeted Sanger sequencing on an ABI Prism™ 3500 DX DNA Sequencer (Thermo Fisher Scientific) following the standard conditions recommended by the manufacturer. Primers were designed (sequences will be available on request), using Primer 3 Program (https://primer3.org/), and Primer-BLAST (https://www.ncbi.nlm.nih.gov/tools/primer-blast/). PCR amplifications and sequencing were performed using the AmpliTaq Gold polymerase and the BigDye Terminator v3.1 Cycle Sequencing kit (Thermo Fisher Scientific), respectively, according to the manufacturer's instructions.

### Statistical analyses

Clinical characteristics were reported as absolute and relative frequencies (i.e. percentages). Age was reported both as median along with interquartile range (i.e. first-third quartiles) and as a binary variable (dichotomized at the cut-off of 55 years). Comparison between categorical variables were performed using Fisher exact test. Prevalence (%) of germline variants in predisposition genes was defined as the number of variants over the total sample and its 95% confidence interval (CI) was computed using the Clopper-Pearson method, based on the binomial distribution. Moreover, exact logistic regression models were performed to quantify the association between the presence (or the type of mutation) of germline variants and the PanC. Risks were reported as odds ratios (OR) along with one-sided 95% CI. Since conditional exact likelihood often did not exist for the model parameters, the OR was provided by an unbiased median estimate, setting the lower or upper bounds of its confidence limits as zero and infinite, respectively. This is the reason for using a 95% one-sided CI alongside all OR estimates. Moreover, time-to-event analysis was performed for overall and progression-free survival providing Kaplan–Meier estimates of survival curves along with log-rank test. P-values < 0.05 was considered for statistical significance. All statistical analyses and plots were performed using R Foundation for Statistical Computing (version 4.0).

## Results

### Characteristics of the study sample

Demographic features, risk factors, family cancer history, and baseline clinical-pathological characteristics of the study population are shown in Table 2. The PanC cohort included 47 patients (median age = 60 yrs; 22 females), and in the CS cohort there were 51 subjects (median age = 63 yrs; 23 females). The 51.1% of PanC patients were less than 55 yrs old, while the 82.4% of CS were greater than or equal to 55 yrs old (p < 0.001). Obese subjects were found in 13% of PanC and in 21.6% of CS; current or past smoking habit was reported by 74.5% of PanC and 66.7% of CS; about 8.5% of PanC and 13.7% of CS were moderate or heavy drinkers; diabetes was present in 34% of PanC and 52.9% of CS, and was classified as early-onset diabetes in 43.8% and 59.3% of the cases and controls, respectively.

**Table 2:** Study population

	PanC (No. 47)	CS (No. 51)
Gender, N (%)		
Male	25 (53.2)	58 (54.9)
Female	22 (46.8)	23 (45.1)
Age, median (IQR)	60 (49–68)	63 (57–68)
< 55 years, N (%)	24 (51.1)	9 (17.6)
≥ 55 years, N (%)	23 (48.9)	42 (82.4)
Body Mass Index, N (%)*		
≤ 30	40 (87)	40 (78.4)
> 30	6 (13)	11 (21.6)
Smoker, N (%)		
No	12 (25.5)	17 (33.3)
Current	17 (36.2)	21 (41.2)
Past	18 (38.3)	13 (25.5)
Alcohol intake, N (%)		
No	16 (34)	16 (31.4)
Moderate	27 (57.5)	28 (54.9)
Heavy	4 (8.5)	7 (13.7)
Diabetes, N (%)		
No	31 (66)	24 (47.1)
Yes	16 (34)	17 (52.9)
Early-Onset	7 (43.8)	16 (59.3)
Family history of cancer, N (%)*		
Pancreas	25 (53.2)	20 (40)
Breast	7 (14.9)	4 (8)
Endometrium	6 (12.8)	0
Colon	5 (10.6)	6 (12)
Melanoma	2 (4.3)	1 (2.0)
Other	19 (40.4)	13 (26)
Pre-operative classification, N (%)*		
Resectable	11 (24.4)	
Locally advanced	15 (33.3)	
Metastatic	19 (42.2)	
Surgery, N (%)*		
No	32 (69.6)	
Yes	14 (30.4)	
Tumor location, N (%)*		
Head	35 (79.5)	
Body/Tail	9 (20.5)	
Tumor stage, N (%)*		
IA	1 (2.3)	
IIB	14 (31.8)	
III	8 (18.2)	
IV	21 (47.7)	
Adjuvant therapy, N (%)*		
No	9 (20.5)	
Yes	35 (79.5)	

^*^Variables with some missing data. PanC: pancreatic cancer; CS: control subjects

A comprehensive family history of cancer among respective first-degree relatives was available for all the 47 PanC: 4 patients (8.5%) had no family history of cancer, 25 (53.2%) had a positive family history for PanC, while 19 patients (40.4%) reported a family history of breast-/ovary-/colorectal-cancer/melanoma, and other 19 (40.4%) had history of tumors not suggestive of cancer predisposition syndromes among their first-degree relatives. Accordingly to criteria for familial PanC or other cancer predisposition syndromes, 2 patients were familial PanC, 34 patients were unselected PanC, and 11 patients were apparently sporadic PanC.

Family cancer history was available for 50/51 CS (98%): 15 subjects (30%) had no family history of cancer among their first-degree relatives, 20 (40%) had positive family history for PanC, while a family history of breast-/ovary-/colorectal-cancer/melanoma or for tumors not suggestive of cancer predisposition syndromes was reported by 11 (22%) and 13 (26%) patients, respectively. Overall, one CS was a subject with familial PanC (i.e. had 2 first-degree relatives affected by PanC), 24 CS did not meet criteria for familial PanC or any cancer predisposition syndromes, and 25 CS had negative family cancer history or a family history of tumors not suggestive of a cancer predisposition syndromes.

### NGS sequencing results

A total of 13 separate sequence runs were performed. The mean number of reads (± standard deviation) produced by each sequence run was 5 + 1.5 M, resulting in a mean number of sequenced bases of sufficient quality (AQ20 = 99% chance correct base called) produced by each sequence run of 971.2 + 291.4 M. The uniformity of the read number for each run was sufficiently high, with 11 of the 13 runs (84.6%) providing more than 5 M reads. The mean depth was 749 ± 211 reads and the mean read length was 194 ± 10 bp. The mean number of the mapped reads (sequencing read mapped on target regions) produced by each sequence run was 4.5 ± 1.4 M.

We considered that only exons (and their adjacent boundaries sequences) with a read depth above 20 reads (20×) for each targeted nucleotide were considered as correctly covered. According to this criterion, our AmpliSeq custom panel allowed us to efficiently explore 765 out of the 775 amplicons; cumulated uncovered regions corresponded to a sequence of 2074 bp including a non-coding sequences of 1703 bp and a coding sequence of 371 bp corresponding to three exons, the 0.6% of the total number exons included in our AmpliSeq panel. An average of 98.9 ± 3.9% of each target region was sequenced with over 20 × coverage. The mean number of reads produced by each sequenced sample was 545.2 ± 273.7 thousand, resulting in a mean number of sequenced bases of sufficient quality (AQ20) of 116.2 + 39.2 M. The mean depth was 753 + 246 reads, and the mean read length was 194 + 9 bp. The mean number of the mapped reads (sequencing read mapped on target regions) was 583 ± 190.6 thousand reads for each sample.

### Germline alterations in PanC cohort

A total of 31 germline variants were identified in 27 out of 47 PanC (57.4%, 95% CI 42.2–71.7), comprising 10 pathogenic-likely pathogenic variants in 8 patients (17.0%; 95% CI 7.6–30.8), 3 variants categorized both as pathogenic-likely pathogenic and VUS in 3 patients (6.4%; 95% CI 1.3–17.5), 15 VUS in 15 patients (31.9%; 95% CI 19.1–47.1), and 3 UVs in 3 patients (6.4%; 95% CI 1.3–17.5).

**Fig. 1:**
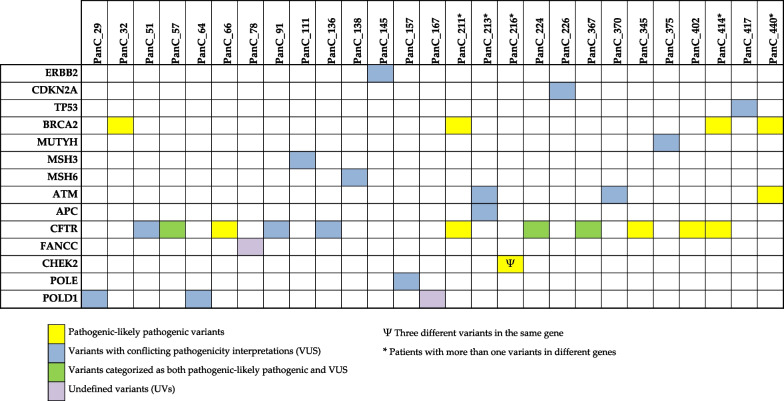
Genes with germline mutations in patients with pancreatic cancer (PanC)

As reported in Fig. 1 and in Additional file 1: Table S1, the identified variants encompassed 14 out of 27 analyzed genes (ERBB2, CDKN2A, TP53, BRCA2, MUTYH, MSH3, MHS6, ATM, APC, CFTR, FANCC, CHEK2, POLE, POLD1), while no mutations were identified in the remaining 13 genes (KRAS, SMAD4, GNAS, RNF43, BRCA1, MLH1, MSH2, PMS2, EPCAM, STK11, PRSS1, SPINK1, FANCC).

Notably, 22 out of the 27 patients with PanC had a single germline variant, while more than one variant was identified in 5 patients: one PanC had 3 variants in CHEK2 gene (c.1209-1233del325, pathogenic; c.1141A > G, VUS; c.1200delG, UVs), three patients had 2 pathogenic-likely pathogenic variants in 2 different genes (BRCA2: c.6037A > T and CFTR: c.1521_1523delCCT; BRCA2: c.8755-1G > A and CFTR: c.3454G > C; BRCA2: 8954_8955delTTinsAA and ATM: c.2502_2503insA), and one other patient had 2 VUS in different genes (APC: c.3049_3051delAAT and ATM: c.5753G > C). In addition, only two of the identified variants, in CFTR (c.1521_1523delCCT, pathogenic) and POLD1 (c.493C > T, VUS), were found more than once, in 2 different patients.

Seven out of the 8 patients with pathogenic-likely pathogenic variants were classified as unselected PanC accordingly to their family cancer history. The remaining one, a 45 years-old patient with the c.1521_1523delCCT pathogenic mutation in CFTR gene, had a negative family cancer history. In details, the 7 patients unselected PanC included: a 51 years-old patient with 3 mutations in CHEK2 (c.1209-1233del325, pathogenic; c.1141A > G, VUS; c.1200delG, UVs) whose mother had died for PanC; two 64 years-old patients with pathogenic-likely pathogenic mutations in BRCA2 and CFTR (c.8755-1G > A and c.3454G > C) or in BRCA2 and ATM (c.8954_8955delTTinsAA and c.2502_2503insA), each with a parent who died for PanC (father and mother, respectively) and one sister affected by endometrial cancer; two PanC with a pathogenic mutation in CFTR (c.3196C > T or c.2417A > G) diagnosed with PanC at the age of 83 and of 49 respectively, and having one first-degree relative who died for PanC (sister and mother, respectively) and another one who died for lung cancer (brother and father, respectively); two PanC were patients younger than 50 years old with no first-degree relatives affected by PanC but with family history positive for breast cancer (mother/sister and sister, respectively) who presented a pathogenic mutation in BRCA2 (c.2244C > G) or two pathogenic mutations in different genes (BRCA2: c.6037A > T and CFTR: c.1521_1523delCCT).

Prevalence of identified variants in PanC patients and CS are shown in Table 3. CFTR (10.6%) and BRCA2 (8.5%) were genes with the highest frequencies of pathogenic-likely pathogenic variants in PanC. Smaller numbers of mutations were observed also in ATM (2.1%) and CHEK2 (2.1%). CFTR gene had also the highest prevalence of pathogenic-likely pathogenic/VUS and VUS (6.4%); pathogenic-likely pathogenic/VUS were also found in ATM and POLD1 (4.3%).

**Table 3:** Prevalence of identified variants in patients with pancreatic cancer and control subjects

	PanC (No. 47)	CS (No. 51)
**Pathogenic-likely pathogenic**		
ATM*	1 (2.1%)	0
BRCA2*	4 (8.5%)	0
CFTR*	5 (10.6%)	1 (2.0%)
CHEK2	1 (2.1%)	0
MUTYH	0	1 (2.0%)
** No. of variants**	**10**	**2**
** No. of individuals with variant(s)**	**8**	**2**
**Pathogenic-likely pathogenic/VUS**		
ATM	0	1 (2.0%)
CFTR*	3 (6.4%)	5 (9.8%)
** No. of variants**	**3**	**5**
** No. of individuals with variant(s)**	**3**	**6**
**VUS**		
APC*	1 (2.1%)	4 (7.8%)
ATM*	2 (4.3%)	5 (9.8%)
BRCA1*	0	2 (3.9%)
CDKN2A	1 (2.1%)	0
CFTR*	3 (6.4%)	5 (9.8%)
CHEK2*	1 (2.1%)	1 (2.0%)
ERBB2	1 (2.1%)	0
FANCC*	0	2 (3.9%)
FANCG	0	1 (2.0%)
MLH1	0	1 (2.0%)
MSH3	1 (2.1%)	0
MSH6	1 (2.1%)	0
MUTYH*	1 (2.1%)	3 (5.9%)
PMS2*	0	1 (2.0%)
POLD1*	2 (4.3%)	0
POLE	1 (2.1%)	2 (3.9%)
TP53	1 (2.1%)	0
** No. of variants**	**15**	**31**
** No. of individuals with variant(s)**	**15**	**25**
**Undefined**		
ERBB2	0	3 (5.9%)
MSH3	0	1 (2.1%)
ATM	0	1 (2.1%)
CFTR	0	1 (2.1%)
FANCC	1 (2.1%)	0
CHEK2	1 (2.1%)	0
POLD1	1 (2.1%)	0
RNF43	0	1 (2.1%)
** No. of variants**	**3**	**7**
** No. of individuals with variant(s)**	**3**	**7**

^*^Genes with mutations found in more than one subject, in subjects with more than one variant in different genes, or in subjects with different variants in the same genes. PanC: pancreatic cancer; CS: control subjects

Bold values indicate the subclasses of variants identified in the study

### Associations between germline variants and PanC

When prevalence of mutations was evaluated by classifying patients based on age and gender, accordingly to the presence of clinical risk factors for the development of PanC, including family history of cancer, and in relation to the characteristics of tumor, no significant associations were found between the prevalence of variants and clinical variables (Additional file 1: Table S2).

We then evaluated the associations between germline mutations and PanC. Only BRCA2 gene showed a significant association, with mutations present in 4/47 PanC (8.5%) compared to 0/51 CS (0%), (OR, 6.07; one-sided 95% CI 1.01–infinity). All the other genes did not shown significant association with PanC (Additional file 1: Table S3). Notably, all the BRCA2 variants identified in PanC had a pathogenic effect. By comparing the clinical and anamnestic characteristics of BRCA2 variants carriers vs. non-carriers, we observed that PanC patients with mutations in BRCA2 were more likely to have a family history for endometrial cancer (50%) compared to the non-carriers (4.3%), p = 0.018 (Additional file 1: Table S4). However, when we assessed the associations between BRCA2 mutation status and tumor features, chemotherapy regimens and clinical outcomes in PanC patients as well as with the overall and progression-free survival. Analyses did not show significant differences between BRCA2 mutations carriers and non-carriers (Additional file 1: Table S5 and Additional file 2: Fig. S1).

## Discussion

Over the recent years, extensive investigation into inherited germline predisposition to PanC has been performed (Wood et al. 2019). Data demonstrate that many individuals who develop PanC in the setting of genetic predisposition lack clinical features or family cancer history typically associated with hereditary cancer-predisposing syndrome. These patients should not be precluded from an opportunity to benefit from genetic testing just because of their personal or family cancer history, and their relatives should not be ruled out form surveillance programs for PanC screening. Accordingly to the provisional clinical opinion of the American Society of Clinical Oncology, all the PanC patients should undergo assessment of risk for hereditary syndromes known to be associated with an increased risk for PanC, even if family history is unremarkable (Stoffel et al. 2019). However, currently guidelines to help clinicians determine which PanC patients may benefit from germline multigene testing are not yet available, and more researches are needed to further understand the association between cancer susceptibility genes and PanC.

The aim of this study was to determine the prevalence of germline mutations in cancer predisposition genes, and to evaluate the association between inherited germline mutations and the presence of PanC. Whether clinical variables, including family cancer history for PanC or any cancer predisposition syndromes, are associated with mutation carrier status was also assessed. To pursue this intent, 27 susceptibility genes were sequenced using a multigene panel in 98 subjects enrolled at a single center. The study population included PanC patients and healthy controls.

Overall, thirty-one germline mutations in 14 genes were identified in 27 PanC. Eight patients had at least one pathogenic-likely pathogenic variants encompassing four main genes: CFTR (5 patients), BRCA2 (4 patients) ATM and CHEK2 (1 patient). Except for the variant c.1521_1523delCTT in CFTR that was found as single variant in a PanC with negative family history for cancer, all the other pathogenic-likely pathogenic variants identified in this study were in patients classified as unselected PanC accordingly to their family anamnesis. The rate of pathogenic-likely pathogenic mutations identified in this study is over the range (3.8% to 11.3%) reported in previous small studies on unselected patients with PanC (Grant et al. 2015; Johns et al. 2017). This may be due to differences in gene panels used among the different studies, and in prevalence and spectrum of germline pathogenic variants among geographically different populations. Of note, when we considered the overall family history for PanC, we observed that pathogenic-likely pathogenic variants were identified in the 20% of patients with a family history positive for PanC (5/25) and in 13.6% of patients without first-degree relatives affected by PanC (3/22). Thus, in line with other reports (Cremin et al. 2020), the use of traditional criteria-based testing would have missed 37.5% (3/8) of patients with pathogenic-likely pathogenic mutations in various genes: BRCA2 (1 patient), CFTR (1 patient), BRCA2/CFTR (1 patient).

In line with the available literature, most of the identified pathogenic-likely pathogenic mutations were in established PanC-predisposition genes (Hu et al. 2016). A recent review pointed out the predominance of deleterious variants in breast and ovarian cancer genes among PanC patients, with BRCA2 and ATM having the highest prevalence of pathogenic mutations in both sporadic and unselected PanC (Astiazaran-Symonds and Goldstein 2021). BRCA2 is an important PanC predisposition gene with germline mutations found in 6% to 10% of patients with familial PanC (Couch et al. 2007), and in about 3.5% of PanC patients unselected for familial history (Holter et al. 2015). In our study, BRCA2 mutations were identified in 4 unselected PanC patients including two patients under the 50 years with positive family history for breast cancer who carried a pathogenic mutation (c.2244C > G and c.6037A > T, respectively), and two 64-years old patients with positive family history for PanC and endometrial cancer who carried a pathogenic-likely pathogenic or a pathogenic mutation (c.8755-1G > A and c.8954_8955delTTinsAA, respectively). In the case–control evaluation the presence of BRCA2 mutations was significantly associated with PanC. In addition, although the small sample size did not allow to make conclusions on the associations with most of the clinical variables, including overall survival and therapies, we found that the presence of BRCA2 mutations was associated with positive family history for endometrial cancer.

Currently, BRCA2 mutation carriers were recommended to undergo screening for PanC, starting from the age of 50, only if they had at least one first‐degree relative (or two relatives regardless of degree) affected by PanC, while no consensus was reached on BRCA2 mutation carriers without family history of PanC (Canto et al. 2013; Goggins et al. 2020). Notably, since none of the BRCA2 mutation carriers identified in this study underwent germline genetic testing before, for all these patients there were no chance to be diagnosed earlier during an active program of surveillance for PanC and to evaluated for the therapeutic implications associated with the mutational status in BRCA2 gene (Roch et al. 2019; Fogelman et al. 2011; Tran et al. 2012). In addition, their unaffected relatives were not genetically tested to identify the mutations carriers to be enrolled into PanC screening protocols (Al-Sukhni et al. 2012; Sud et al. 2014). In addition, since BRCA2 is a gene for which current guidelines provide specific screening and interventions to reduce the risk of associated cancers, the lack of determination of BRCA2 germline variants in both patients and their relatives has also limited the great potential to facilitate extra-pancreatic cancer prevention in these subjects (Berek et al. 2010; Hartmann et al. 2001; Al-Sukhni et al. 2008). Overall, albeit under-powered this single center study underlined the potential remarkable implications that contributions of predisposition genes to the risk of PanC could have for patients and their blood relatives.

As to ATM, the association between deleterious variants in this gene and PanC has been reported in patients with familial PanC and in those unselected for family history (Hu et al. 2018; Roberts et al. 2012, 2016). However, the lifetime risk of PanC in carriers has not been well defined. The pathogenic mutation in ATM gene identified in this study (c.2502_2503insA) was found in a patient with family history positive for PanC and endometrial cancer, who presented also the BRCA2 pathogenic variant c.8954_8955delTTinsAA. Although we did not found a significant association between ATM and PanC, based on this observation an effect of ATM on cancer germline predisposition not independent of BRCA2 might be supposed.

Although hereditary pancreatitis genes are often not included in multigene panels, probably due to a biologically different mechanism of these genes in PanC development, the presence of mutations in CFTR has been associated with a modest increased risk for PanC, with affected appear to be diagnosed at a younger age, especially among smokers (McWilliams et al. 2010, 2005; Wilschanski and Durie 2007; Durno et al. 2002; Cazacu et al. 2018). It has been also reported that CFTR mutation carriers with other risks for the development of PanC, such as a positive family history for the disease, should undergo screening protocols (Rittenhouse et al. 2011). In our study, the most common CFTR pathogenic mutation associated with PanC (c.1521_1523delCCT) was identified in two PanC under the 50 years without family history for PanC who were current or past heavy smokers, and in one CS with family history positive for PanC; other pathogenic-likely pathogenic mutations in CFTR gene (c.3454G > C, c.3196C > T, c.2417A > G) were identified in 3 PanC with positive family history for PanC diagnosed at the age of 64, 83 and 49, respectively, with the two older patients reporting a past heavy smoking habit. CFTR showed also the highest prevalence of pathogenic-likely pathogenic/VUS and VUS, occurring in 3 patients. Overall, despite the high prevalence of variants identified in CFTR, and in line with other studies on small study population (Matsubayashi et al. 2003), we did not found significant association between variations in CFTR gene and PanC risk suggesting that further studies are needed to verify the contribution of this gene to the development of PanC.

Further studies are also required to define the contribution of CHEK2 and POLD1 to the PanC susceptibility. Herein, 3 different variants in CHEK2 (1 pathogenic, 1 VUS and 1 UVs) were identified in a 51 year-old patient with family history of PanC. CHEK2 is a multi-organ cancer predisposition gene playing an important role in cell cycle regulation and DNA damage repair, two processes involved in cancer development and response to treatment (Cybulski et al. 2004). Recently, the Cancer Genome Atlas (TCGA) Research Network reported that CHEK2 mutations were observed in 0.7% of PanC patients (Vittal et al. 2021). CHEK2 mutations are rare and associated with modest penetrance, so very large sample sizes are needed to identify significant relative risks (Cybulski et al. 2004); in addition, the presence of many VUS makes sometimes it difficult to reach a conclusive genetic interpretation. In line with these observations, our data did not allow to make conclusions about germinal variants in CHEK2 and the risk for PanC.

POLD1 has been recently recognized as hereditary cancer-predisposing genes (Magrin et al. 2021). Germline pathogenic variants in proofreading polymerases predispose mainly to multiple colorectal adenomas and carcinomas, but evidence of extra colonic tumors including endometrial, brain, breast, ovarian, stomach, pancreas, and skin tumors, have been also reported for mutation carriers (Mur et al. 2020; Buchanan et al. 2018). In this study, 2 first-degree relatives (brother and sister) diagnosed with PanC at the age of 65 and 62, respectively, presented a single germline VUS, namely c.493C > T in POLD1 gene. These patients had a family history positive for endometrial cancer (mother and sister), throat cancer (father), gastric cancer (brother), and prostate cancer (brother). This variant is located outside the exonuclease domain, and its role in the ClinVar database had conflicting interpretation of pathogenicity. Thus, although the limited supporting evidence at this time for the clinical significance of this variant, based on the cancer family history and on the germline mutational pattern identified, our data suggested that further examinations will clarify the clinical significance of this VUS in POLD1 gene.

There are some limitations to the study. The sample size certainly represents the main limitation of our investigation, since overall larger populations are needed when studying rare pathogenic mutations. In addition, the gene panel used in this study did not include all the possible cancer predisposition genes, implying that other genes may emerge as potential candidate for genetic testing in PanC patients. Therefore, further large-scale replication of our study should be evaluated for future investigations.

## Conclusions

Accordingly to the recommendations of the National Comprehensive Cancer Network and to the provisional clinical opinion of the American Society of Clinical Oncology (Stoffel et al. 2019; Tempero et al. 2017), this study encourages germline testing for all the newly diagnosed PanC. Our multigene panel evaluation uncovered germline variations of four main cancer susceptibility genes in PanC patients unselected for family cancer history. We confirmed the significant association between pathogenic-likely pathogenic variants in BRCA2 and the increase of PanC risk, with variants identified more frequently among patients with a positive family history of endometrial cancer. Our data also encouraged further studies on ATM, CFTR, CHEK2 and POLD1 aimed to determine the contribution of these genes to PanC genetic predisposition. Overall, in order to reduce the number of underdiagnosed carriers of mutation in cancer susceptibility genes, germline testing should be offered to all the patients PanC without clinical evidences of inherited component for PanC or other cancer predisposition syndromes.

## Supplementary Information

**Additional file 1: Table S1**. Complete list of the identified germline variants. **Table S2**. Association between variants prevalence and clinical variable within each group of subjects. **Table S3**. Associations between the presence of germline mutations and pancreatic cancer. **Table S4**. Association between the presence of BRCA2 mutation and clinical variables. **Table S5**. Association between the presence of BRCA2 mutation and tumor features and therapies in pancreatic cancer.

**Additional file 2: Figure S1**. Kaplan–Meier survival curves, along with p-values from log-rank test, showing the overall survival (right panel) and the disease survival free (left panel) probability between pancreatic cancer patients with or without BRCA2 mutations.

## Data Availability

The detailed data presented in this study are available from the corresponding authors on reasonable request.

## Abbreviations

PanC: Pancreatic cancer
CS: Control subjects
NGS: Next Generation Sequencing
VUS: Variant of uncertain significance
UVs: Uncategorized variants
CI: Confidence interval
OR: Odds ratio
