# Supplementary figures and images for: Evaluation of inherited germline mutations in cancer susceptibility genes among pancreatic cancer patients: a single-center study
Source: Mol Med. 2023 Jan 30;29:14. doi: 10.1186/s10020-023-00600-1 (PMC9885574; doi:10.1186/s10020-023-00600-1)

**SUPPLEMENTARY FIGURE 1**

**
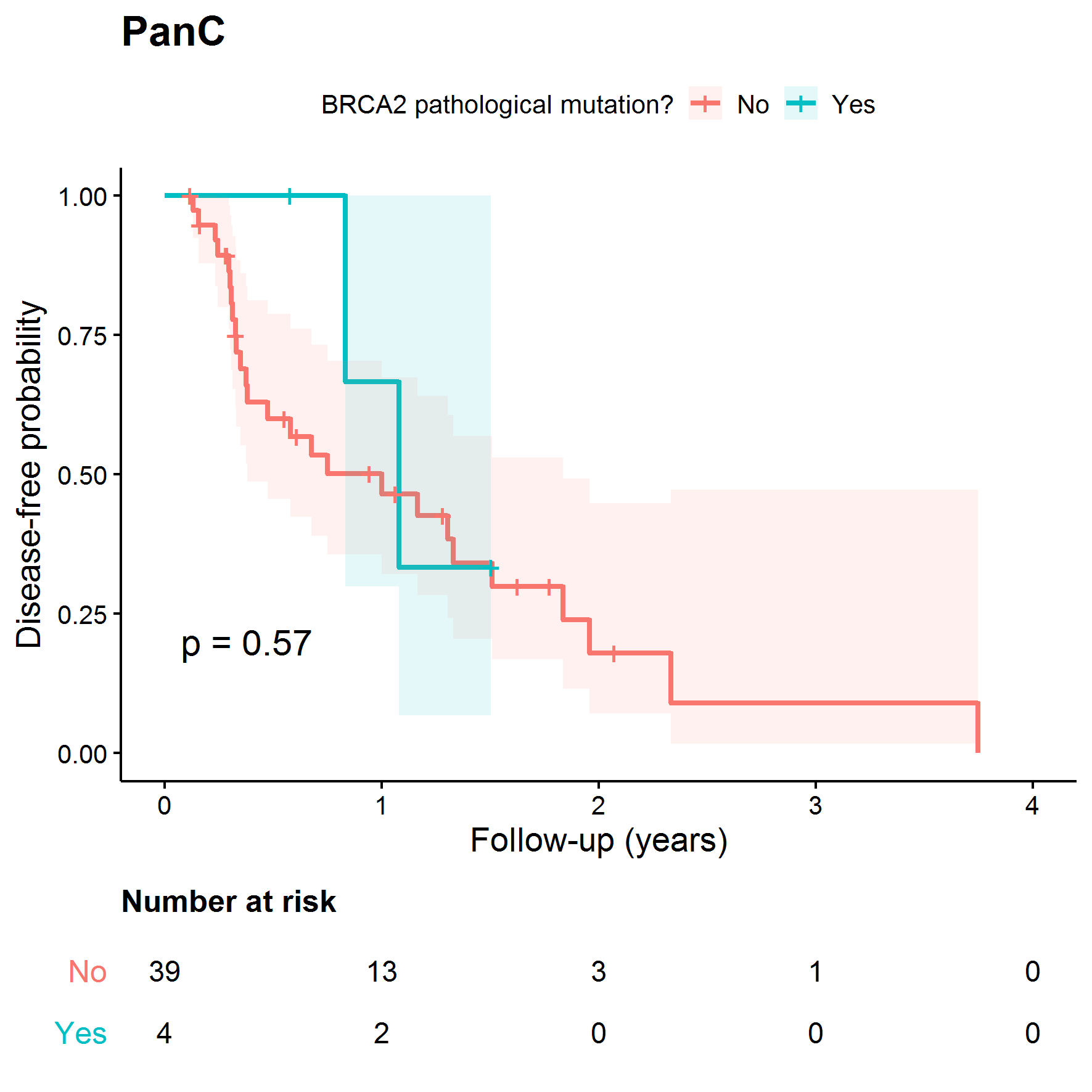

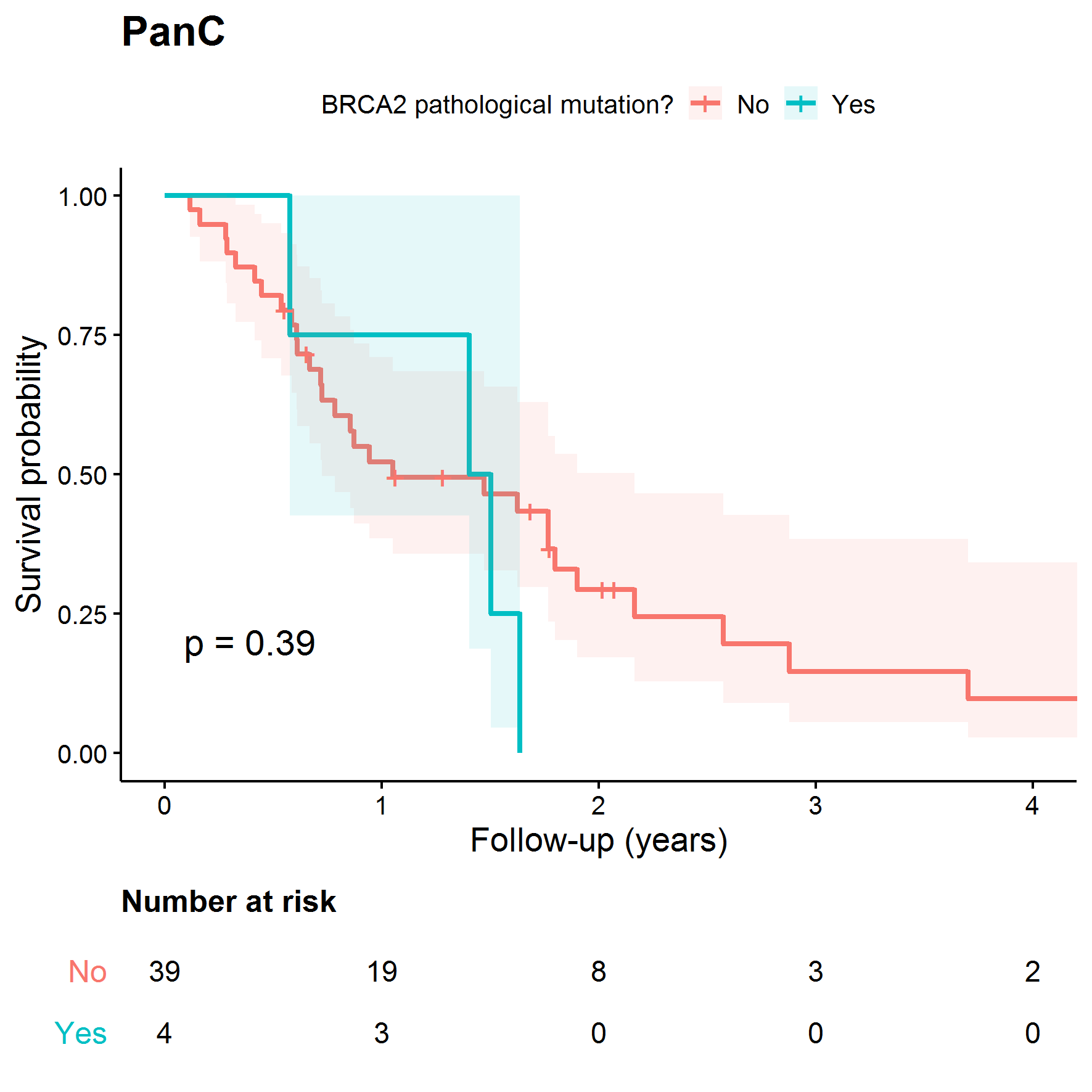
**

Supplement: Supplementary file 2 — Additional file 2: Figure S1. Kaplan–Meier survival curves, along with p-values from log-rank test, showing the overall survival (right panel) and the disease survival free (left panel) probability between pancreatic cancer patients with or without BRCA2 mutations. [file 10020_2023_600_MOESM2_ESM.docx]
